# Retraction: Berberine and cinnamaldehyde together prevent lung carcinogenesis

**DOI:** 10.18632/oncotarget.28577

**Published:** 2024-08-05

**Authors:** Mingjing Meng, Shengnan Geng, Zhenhua Du, Jingjing Yao, Yaqiu Zheng, Zibo Li, Zhenzhen Zhang, Jiahuan Li, Yongjian Duan, Gangjun Du

**Affiliations:** ^1^Institute of Pharmacy, Pharmacy College of Henan University, Jinming District, Kaifeng, Henan Province 475004, China; ^2^Department of Oncology, The First Hospital Affiliated to Henan University, Kaifeng, Henan Province 475001, China; ^*^These authors contributed equally to this work

**This article has been retracted:** This article contains duplicated tumor images found in previously published papers, and some overlaps of IHC images (Figure 5) and wound healing images (Figure 8) representing different experimental conditions. Specifically, an tumor image in Figure 1C also appears in Figure 1C of an earlier published paper [1]. A second tumor image in Figure 1C is also duplicated in Figure 2C of a second earlier paper [2]. Therefore, the Editorial decision was made to retract this paper. All authors agreed with the decision.

Original article: Oncotarget. 2017; 8:76385–76397. 76385-76397. https://doi.org/10.18632/oncotarget.20059
